# Genetic mapping reveals a candidate gene *CmoFL1* controlling fruit length in pumpkin

**DOI:** 10.3389/fpls.2024.1408602

**Published:** 2024-05-23

**Authors:** Yimei Zhou, Meng Zhao, Qinghui Shen, Mengyi Zhang, Chenhao Wang, Yutong Zhang, Qinrong Yang, Yongming Bo, Zhongyuan Hu, Jinghua Yang, Mingfang Zhang, Xiaolong Lyu

**Affiliations:** ^1^ Laboratory of Germplasm Innovation and Molecular Breeding, College of Agriculture and Biotechnology, Zhejiang University, Hangzhou, China; ^2^ Hainan Institute of Zhejiang University, Sanya, China; ^3^ Ningbo Weimeng Seed Company, Ningbo, China; ^4^ Key laboratory of Horticultural Plant growth, Development and Quality Improvement, Ministry of Agriculture, Hangzhou, China

**Keywords:** pumpkin, fruit length, BSA-seq, *CmoFL1*, E3 ubiquitin ligase

## Abstract

Fruit length (FL) is an important economical trait that affects fruit yield and appearance. Pumpkin (*Cucurbita moschata* Duch) contains a wealth genetic variation in fruit length. However, the natural variation underlying differences in pumpkin fruit length remains unclear. In this study, we constructed a F_2_ segregate population using KG1 producing long fruit and MBF producing short fruit as parents to identify the candidate gene for fruit length. By bulked segregant analysis (BSA-seq) and Kompetitive Allele-Specific PCR (KASP) approach of fine mapping, we obtained a 50.77 kb candidate region on chromosome 14 associated with the fruit length. Then, based on sequence variation, gene expression and promoter activity analyses, we identified a candidate gene (*CmoFL1*) encoding E3 ubiquitin ligase in this region may account for the variation of fruit length. One SNP variation in promoter of *CmoFL1* changed the GT1CONSENSUS, and DUAL-LUC assay revealed that this variation significantly affected the promoter activity of *CmoFL1*. RNA-seq analysis indicated that *CmoFL1* might associated with the cell division process and negatively regulate fruit length. Collectively, our work identifies an important allelic affecting fruit length, and provides a target gene manipulating fruit length in future pumpkin breeding.

## Introduction

The fruit is an important organ in horticultural crops, which has a rich variety of shapes and sizes. Fruit length (FL) is a key indicator of fruit yield and appearance quality, directly affects fruit shape and size, and also exhibits a broad range of variation, thus emphasizing its importance in breeding program. Previous studies have showed that the fruit length and fruit shape were controlled by a single gene in some populations (Dou et al., 2018; Cheng et al., 2021; Ma et al., 2022), while extensive studies have confirmed that the fruit length and shape were commonly modulated by both quantitative trait loci (QTLs) and some environmental factors (Wang et al., 2020b, c). The genetic analysis of quantitative traits using mixed inheritance model with a major gene plus a polygene have elucidated the genetic rules regulating fruit length and fruit size in some crops (Ren et al., 2022; Yan et al., 2023). The genetic models that control fruit length were different in different crops.

Many important genes and quantitative trait loci (QTLs) regulating fruit length have been reported in different horticultural crops. In tomatoes, *OVATE* and *SUN* control fruit elongation. *OVATE* encodes a family of OVATE proteins (OFPs), which are negative growth regulators that control fruit length by blocking transcription, leading to the appearance of pear-shaped fruit in tomato (Liu et al., 2002; van der Knaap et al., 2014). *SUN* encodes a calmodulin-binding protein in the IQ-Domain (IQD) family that regulates plant growth and fruit shape by altering the way cells divide (Wu et al., 2011). In pepper, fruit length was significantly increased by down-regulation the expression of *CaOvate* through VIGS (Tsaballa et al., 2011). The gene *Smechr0301963*, which belongs to the *SUN* gene family, was predicted to be a key candidate gene for eggplant fruit length regulation (Wei et al., 2020). *CmCLV3*, which determines carpels number, is also thought to influence fruit shape in the andromonoecious genetic context in melon (Liu et al., 2020). In addition, overexpress the CRABS CLAW family gene *CmCRC* in melon can affect fruit elongation (Switzenberg et al., 2015). Depending on different genetic backgrounds, cucumber fruit length can be controlled by different single inherited sites, such as *CsSUN*, *CsTRM5*, *CsSF1*, *CsFUL1*, *CsSF2* genes (Pan et al., 2017; Wu et al., 2018; Xin et al., 2019; Zhao et al., 2019; Zhang et al., 2020). In watermelon, using materials with different genetic backgrounds, a major QTL controlling fruit shape was detected on chromosome 3, and the homologous gene *Cla011257* of *SUN* was found in this region (Sandlin et al., 2012; Dou et al., 2018; Legendre et al., 2020).

Pumpkin (*Cucurbita moschata* Duch) is one of the most important horticultural crops of cucurbits, which also serve as mainstays of diet to provide significant sources of nutrition. Pumpkin contains a wealth genetic variation in fruit shape and size, and fruit length exhibits great difference, varying from 5 cm to more than 100 cm (Oliveira et al., 2016; Paris, 2018; Ezin et al., 2022). With the development of pumpkin genomics (Montero-Pau et al., 2017; Sun et al., 2017), the mapping of fruit-related genes has been greatly promoted, and many QTLs that control fruit length and shape have been identified. In *Cucurbita pepo*, QTL mapping analysis was conducted on the size and shape of immature and mature fruits, and major QTLs (*IFSh_3, IFLe_3, IFWi_3, MFSh_3, MFLe_3*, and *MFWi_3*) were detected related to fruit shape (Esteras et al., 2012; Montero-Pau et al., 2017). Three QTLs with the total PVE of 20.25% were detected for FSI in pumpkin, and the *fsi2.1* and *fsi6.1* were overlapped with *fl2.1* and *fl6.1*, respectively, suggesting that fruit length plays a major role in determining fruit shape (Han et al., 2022).

In this study, to anchor the candidate gene of pumpkin fruit length, we developed F_2_ population derived from the KG1 (extra-long pumpkin) and MBF (short-fruit pumpkin) hybrid. The fruit length of KG1 is about 107.13 cm, while that of MBF is about 12.25 cm. Through BSA-seq analysis and KASP approaches, we obtained a 50.77 kb candidate region on chromosome 14 associated with the fruit length. By sequence variation, gene expression and promoter activity analyses, we finally revealed a candidate gene *CmoFL1* controlling fruit length, which encodes E3 ubiquitin ligase. Our finding will help to clarify the genetic rules of fruit length regulation and provide valuable genetic resources for the production and breeding of new varieties of pumpkin with excellent fruit type.

## Materials and methods

### Plant material, statistical and genetic analyses

Two lab-preserved pumpkin accessions (KG1 and MBF) were selected and used to construct the fourth-generation population for genetic analysis of fruit length. KG1 produces a long fruit, while MBF produces a short fruit. F_1_ plants were obtained by crossing KG1 (P_1_) as female parent and MBF (P_2_) as male parent in 2019, and the F_2_ population was obtained by self-crossing F_1_ plants. Seedlings of F_2_ were raised in a greenhouse in Hangzhou City, China, in the spring of 2020. According to pumpkin cultivation technology, all plants adopt unified water fertilizer and pest management methods to ensure the same growth and development environment of individual plants. The pumpkin fruit length (cm) was measured with a tape and recorded on the day of flowering and at fruit maturity.

SPSS 23 was used to statistically analyze the phenotypic data of four generations of pumpkin populations, including the average, standard deviation, skewness and kurtosis. The fruit length was expressed as “mean ± standard deviation (mean ± SD)”, and the significance was analyzed by ANOVA. P < 0.05 indicated that there was a significant difference.

Genetic analysis was performed using R software package SEA v2.0.1(https://cran.r-project.org/web/packages/SEA/index.html). Akaike’s information criterion (AIC) value was calculated in each model to identify the existence of major genes affecting quantitative traits.

### Cytological analysis

To compare the cytological characteristics of fruits from two parents, the ovaries of two parents at different stages before flowering and the fruits 35 days after pollination were picked respectively. Pulp with a size of about 1cm³ was cut from the part between pumpkin peel and heart cavity and fixed in FAA fixative solution. The fruit was embedded in paraffin (Surgipath), and then used to generate 8-μm–thick sections in longitudinal directions using a model no. RM2235 microtome (Leica), before staining with fast green stain and imaging, the cell characteristics of ovary and fruit of the two parents at different stages were observed under microscope.

### DNA extraction and library construction

The genomic DNA of the parents, and of the F_1_ and F_2_ populations, were extracted from fresh leaves of young seedlings using Cetyltrimethylammonium Bromide (CTAB) method (Porebski et al., 1997). The concentration and purity of the extracted DNA were determined by agarose gel electrophoresis and Nanodrop 2000 (Thermo Fisher Scientific, USA). 20 long-fruit F_2_ individuals (FL > 45 cm) and 20 short-fruit F_2_ individuals (FL < 20 cm) were mixed equally to construct L-pool and S-pool, respectively. Truseq Nano DNA HT Sample preparation Kit (Illumina) was used to generate sequencing libraries, and the Illumina HiSeq PE150 platform was used to sequence these libraries. The quality of the sequencing data was determined using FASTQC (Brown et al., 2017). The QC standard pipelines were employed as described in the literature (Lyu et al., 2022).

### BSA-seq analysis

The *Cucurbita moschata* genome v1 (http://cucurbitgenomics.org/ftp/genome/Cucurbita_moschata/v1/) was used as a reference genome. The sequencing data were compared with the reference genome to analyze the sequencing depth and coverage. SAMtools software was used to convert BAM files. Single nucleotide polymorphism (SNP) variant calling was conducted using the GATK software with the Unified Genotype function and Variant Filtration (McKenna et al., 2010). The annotations of SNPs were performed using ANNOVAR software (Wang et al., 2010) based on the GFF3 files of the reference genome. The homozygous SNPs/InDels between two parents (KG1 and MBF) were extracted from the VCF files. In order to analyze the frequency differences of SNPs/InDels in offspring, the SNP indexes were calculated based on the read depth information for homozygous SNPs (Takagi et al., 2013) in the two offspring pools (L-pool and S-pool). Then, we calculated the ratio of different reads in the total number, which represented the SNP/InDel indexes of the base sites. We used sliding window methods to calculated the SNP/indel indexes of the whole genome and selected 1Mb as window, 1Kb as step size. The difference in the SNP/indel indexes between two pools was calculated as the Δ (All- index), 95% and 99% confidence levels were selected as the threshold line. Moreover, the G prime method was performed to calculate the G’ value by using the QTLseqr package in R (Mansfeld and Grumet, 2018).

### Fine mapping through KASP markers

To narrow down the genetic interval for fine mapping and to verify the accuracy of BSA-seq, we employed 18 pair of KASP primer combinations (FAM, HEX, Common) as markers based on SNPs/InDels generated from BSA-seq (**Supplementary Table S1**). The KASP markers were used for genotyping verification in both parents and part of F_2_ population, and the primers with obvious genotyping effect were used for single plant polymorphism screening in all F_2_ population. The KASP protocol was utilized as follows: Stage 1: preread stage, 30°C for 1 min; Stage 2: hold stage 94°C for 15 min; Stage 3: PCR stage (touchdown), 94°C for 20 s, 61°C for 1 min (decrease pf -0.6°C), recycling for 9 times (a total of 10 cycles), achieving a final annealing temperature of 55°C; Stage 4: PCR stage, 94°C for 20 s, 61°C for 1 min, recycling for 25 times; and Stage 5: postread stage, 30°C for 1 min. After the amplification, an ABI PRISM 7900HT (Applied Biosystems) was used to detect the fluorescence signal and validate the classification.

### qRT-PCR analysis for candidate gene

The length of the ovary of parents from the formation of the ovary to the day of flowering were measured and recorded, and 5 biological replications for each variety were observed. Based on this, judge which period the ovaries of different lengths belong to. We took the ovaries of KG1 and MBF -12, -8, -4 and 0 days after anthesis (DAA), and stored them at - 80°C for further analyses.

The amplification primers were designed using Primer Premier 6.0 program based on relevant gene sequences from *Cucurbita Moschata* genome database (http://cucurbitgenomics.org/organism/9). The Easy RNA Kit (Easy-Do, Hangzhou, China) was used to extract RNA from the fruits of both parents before flowering according to the manufacturer’s instructions. The cDNA was synthesized from 1 ng of the total RNA using ReverTra Ace qPCR RT Master Mix (Toyobo, Japan). We used the TOROGreen qPCR Master Mix (Toyobo, Japan) for qRT-PCR reactions by Thermo Fisher ABI Stepone Plus real-time fluorescence quantitative PCR. The relative expression levels of candidate genes were determined by using the 2^−ΔΔCt^ comparative method (Livak and Schmittgen, 2001), with gene *ACTIN* as an internal control. All analyses were conducted with three biological and technical replications. The primer sequences used for the above studies are listed in **Supplementary Table S2**.

### Promoter activity analysis

Promoter activity of *CmoFL1* was analyzed using tobacco transient expression assay. The promoter sequence of *CmoFL1* from MBF and KG1 were cloned into upstream of the luciferase (LUC) coding sequence in the pGreen II 0800-LUC vector. The Renilla luciferase (REN) gene, driven by 35S promoter, was used as an internal control. By using Agrobacterium-mediated transformation, the plasmids were transformed into the leaves of tabacco (*N. benthamiana*). Grown two days in greenhouse, the relative luciferase activity LUC/REN was measured using the Dual-Luciferase Reporter Gene Assay Kit (Yeasen, Shanghai, China).

### RNA-seq analysis

Total RNA was extracted from long-fruit KG1 and short-fruit MBF at -12 and 0 DAA. RNA sequencing of 1 µg RNA was performed using Illumina TruseqTM platform. Each stage had three biological replications. After filtering raw data and checking the sequencing error rate and G/C ratio, we obtained clean reads. The clean reads were mapped onto the Cucurbita moschata reference genome (http://cucurbitgenomics.org/ftp/genome/Cucurbita_moschata/v1/) using HISAT2 software. FPKM (fragments per kilobase of transcript per million mapped reads) values were calculated to estimate gene expression levels by StringTie software (Pertea et al., 2015). Transcripts with a fold change (FC) ≥ 1 and Padj ≤ 0.05 were identified as differentially expressed genes (DEGs) via DESeq2 software (Love et al., 2014).

## Results

### Phenotypic differences of fruit length in two parental lines

**Figure 1:**
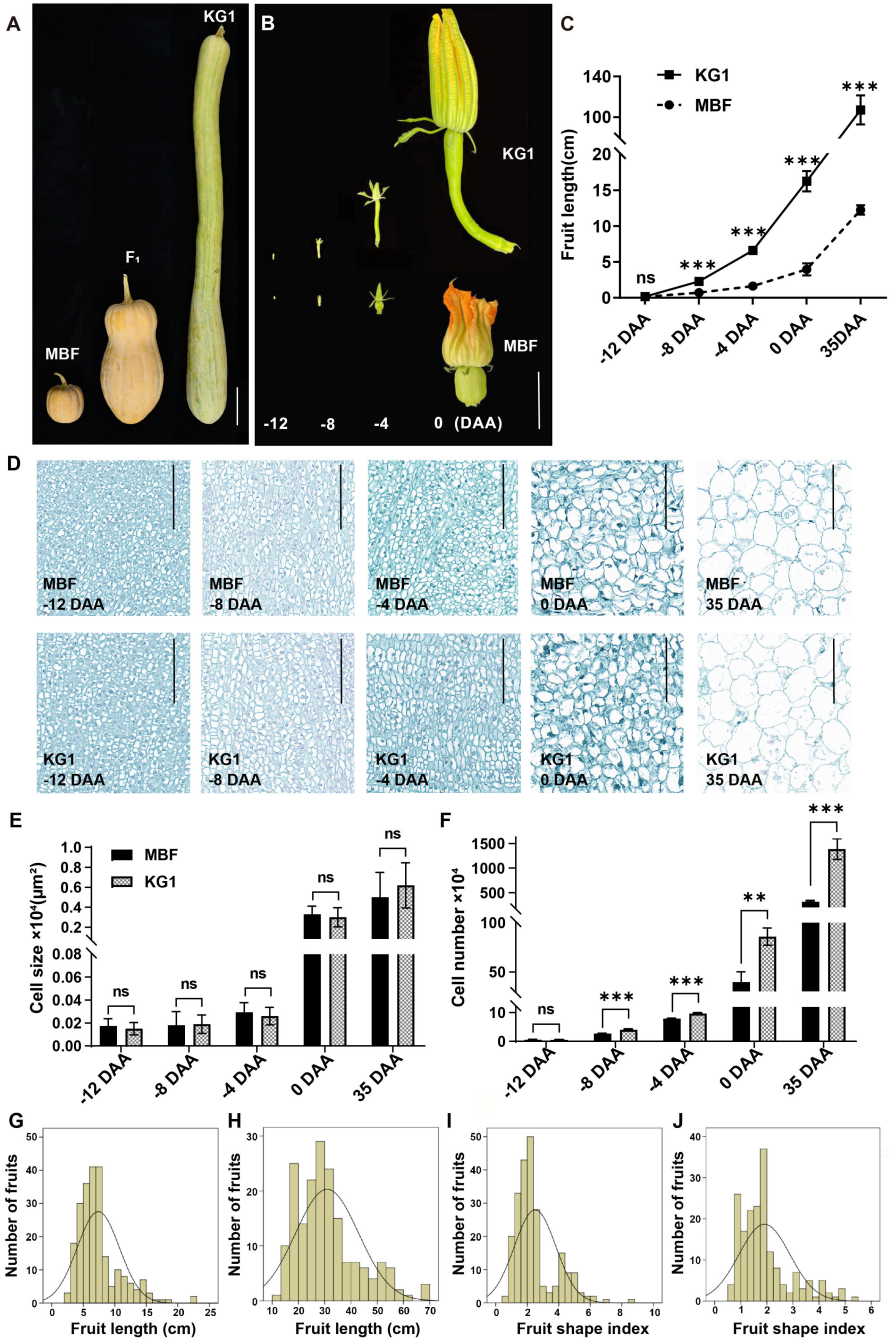
Phenotypic differences of fruit length in two parents. **(A)** The mature fruits of MBF, KG1 and F_1_ generation. Scale bar, 10cm. **(B)** Dynamic changes of ovary morphology between KG1 and MBF from 12 days before anthesis to the day of anthesis. Scale bar, 5 cm. **(C)** The variations in fruit length of KG1 and MBF during ovary development. **(D)** Longitudinal section of fruit cells of MBF and KG1 at different developmental stages (-12 DAA, -8 DAA, -4 DAA, 0 DAA, 35 DAA). Scale bars, 100 µm. **(E)** Average cell size at different developmental stages of MBF and KG1. **(F)** Average number of cells at different developmental stages of MBF and KG1. **(G, H)** Frequency distribution of FL among F_2_ individuals at 0 DAA **(G)** and 35 DAA **(H)**. **(F)**. **(I, J)** Frequency distribution of FSI among F_2_ individuals at 0 DAA **(I)** and 35 DAA **(J)**. **(P<0.01), ***(P<0.001) indicates significant difference by Student’s T-test, while ns shows non-significant different.

To investigate the inheritance pattern of the fruit length trait in pumpkin, we developed F_1_ and F_2_ populations by crossing KG1 (long fruit) and MBF (short fruit). The average FL of KG1 at maturity is 107.13 ± 14.33 cm, which is significantly longer than that of MBF, with an average FL of 12.25 ± 0.65 cm (**Figure 1A**; **Supplementary Table S3**). There was no significant difference in fruit diameter (FD) between the two parents. The average FD of KG1 was 12.98 ± 0.83 cm and that of MBF was 12.85 ± 0.70 cm and the fruit shape index (FSI) of KG1 and MBF were 8.32 ± 1.52 and 0.96 ± 0.07, respectively (**Supplementary Table S3**). We also investigate the fruit length between the two parents during ovary development, and found that the fruit length of KG1 increased rapidly before anthesis and was significantly longer than that of MBF after -12 DAA (**Figures 1B, C**).

Previous studies have showed that the organ shape and size were directly related to cell number and size (Pan et al., 2020). In order to investigate the difference of cell structure between two parents, we analyzed the paraffin sections from fruits of parents at different developmental stages (-12 DAA, -8 DAA, -4 DAA, 0 DAA, 35 DAA) (**Figure 1D**). Observation on the slices showed that there was no significant difference in cell size and morphology between MBF and KG1 during ovary development (**Figure 1E**). The cell number of MBF was consistently less than that of KG1 after -12 DAA, and the gap of cell number became larger with the development of ovary. The cell number of two parents showed a highly significant difference at -8, -4, 0 and 35 DAA (**Figure 1F**). These results revealed that the length difference between KG1 and MBF might mainly came from the number of cells.

### Inheritance pattern of fruit length in pumpkin

Since there was a significant difference in fruit length between two parents before pollination, we measured the FL and FSI of F_2_ individuals at both 0 DAA and 35 DAA (at maturity) (**Supplementary Table S3**). The correlation analysis of FL and FSI showed highly significant correlations between FL and FSI at 0 DAA and 35 DAA (**Supplementary Table S4**), indicating that before flowering, the shape of pumpkin ovary has been formed and remained stable during fruit growth. Furthermore, FL and FSI showed a wide range of variation in F_2_ population. At 0 DAA and 35 DAA, FL varied in the range of 2.70–22.40 cm and 13.00–69.50 cm, respectively, while FSI varied in the range of 0.17–8.61 and 0.56–5.30, respectively (**Supplementary Table S3**). The distribution of FL and FSI were consistent with a positively skewed distribution (**Figures 1G-J**), which indicated that the pumpkin fruit length was a quantitative trait controlled by multiple genes. In addition, the average FL and FSI of F_1_ fruits at maturity were 31.88 ± 4.01 cm and 1.96 ± 0.24 cm, respectively (**Supplementary Table S3**).

**Figure 2:**
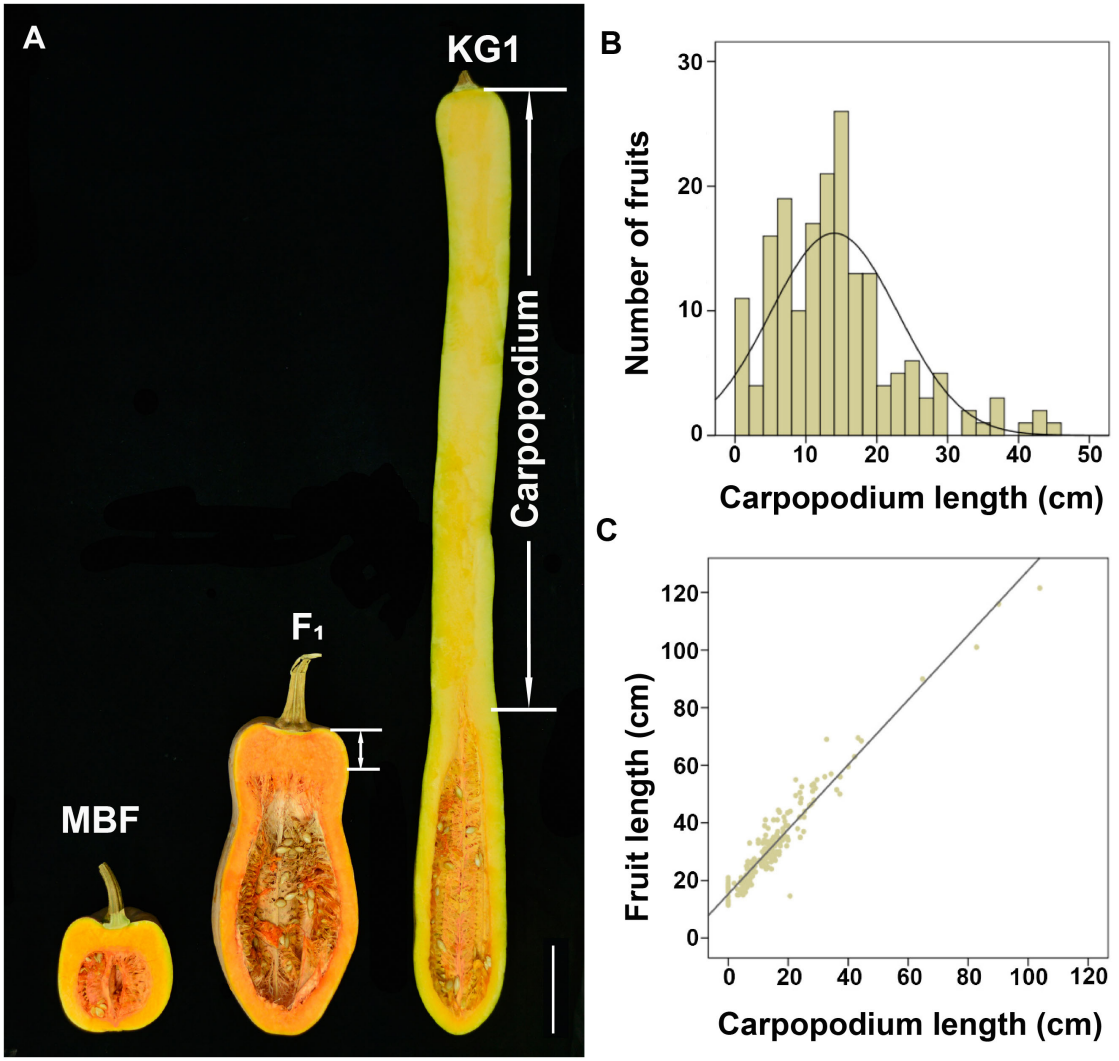
The correlation between fruit length and carpopodium length **(A)** Longitudinal section of mature fruit of MBF, KG1 and F_1_ generation. Scale bar, 10cm. **(B)** Frequency distribution of mature fruit carpopodium length among F_2_ individuals. **(C)** The correlation between fruit length and carpopodium length in F_2_ population.

Moreover, we also found a significant difference of carpopodium length between two parents. KG1 has a carpopodium of about 85.43 cm, accounting for 79.74% of the total fruit length, while MBF has no carpopodium (**Figure 2A**). The carpopodium length of mature fruits were also measured in F_2_ population, which was consistent with the variation of fruit length, and also displayed a skewed distribution in F_2_ population (**Figure 2B**). To confirm the relevance between fruit length and carpopodium length, the correlation analysis was performed, which showed a significant positive correlation between them (**Figure 2C**), suggesting that the carpopodium length might determine the fruit length in pumpkin.

Genetic models for fruit length and carpopodium length were calculated based on the phenotypic data of P_1_, P_2_, F_1_, F_2_ populations. According to the lowest or relatively lower Akaike’s information criterion (AIC) value and no significant (p < 0.05) parameter in the good-of-fit test (**Supplementary Tables S5, S6**), the optional genetic model for fruit length and carpopodium length was 1MG-A, indicating that a single major gene with an additive effect might be responsible for the pumpkin fruit length and carpopodium length.

### BSA-seq analysis anchors a candidate region on chromosome 14 for fruit length

**Figure 3:**
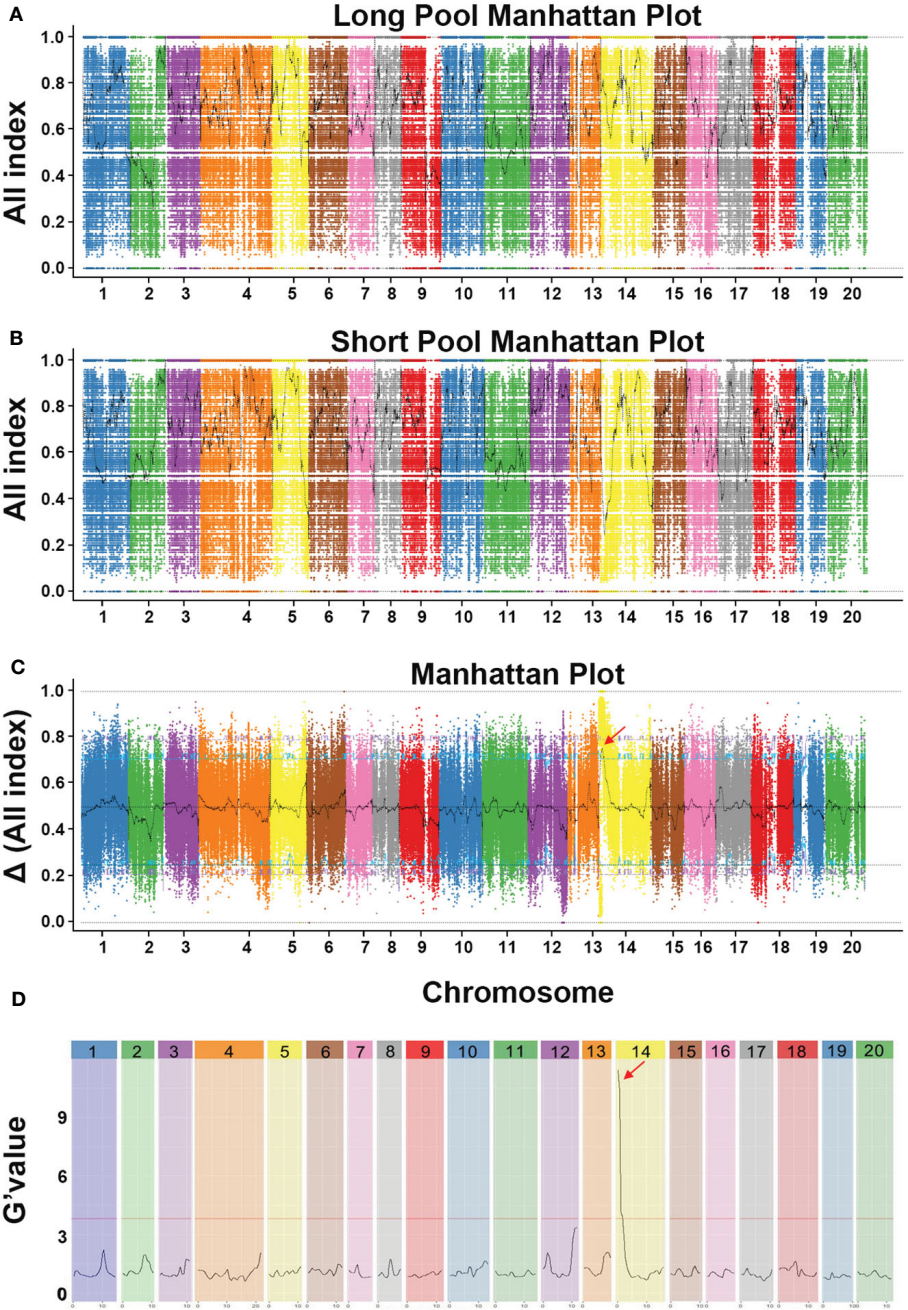
BSA-seq analysis results of the pumpkin fruit length. **(A)** Graph of the All-index of the long pool. **(B)** Graph of the All-index of the short pool. **(C)** Graph of Δ All index values used for the association analysis. The x-axis indicates the 20 pumpkin chromosomes, and the y-axis indicates its All-index value. Different chromosomes are distinguished by different colors. The black curve indicates the filter All index and Δ All index. The blue line represents the 95% threshold line and the purple line represents the 99% threshold line. **(D)** Major quantitative trait loci for pumpkin fruit length identified by QTLseqr.

To anchor the candidate QTL responsible for fruit length, we conducted BSA-seq based on genome resequencing data analysis. Among the 231 F_2_ individuals, 20 long-fruit individuals (FL > 45 cm) and 20 short-fruit F_2_ individuals (FL < 20 cm) were mixed equally to construct L-pool and S-pool, respectively. Then four pools (MBF, KG1, L-pool and S-pool) were sequenced on the Illumina HiSeq™PE150 platform. After filtering low-quality and short reads, a total of 3.53 Gb and 3.65 Gb clean reads was generated from KG1 and MBF, respectively, while 6.57 Gb and 6.80 Gb clean reads were generated from the L-pool and S-pool, respectively (**Supplementary Table S7**). The filtered reads of each sample were aligned to the reference genome of *Cucurbita moschata* genome v1 and the mapping rate was 97.54% ~ 98.31%, the average depth was 10.74× ~ 19.7× (**Supplementary Table S7**). In total, 1,898,813 SNPs and 285,428 InDels were detected after removing SNPs and InDels with low coverage or discrepancies between the parental lines and the bulks. For identifying SNPs/InDels associated with the fruit length trait, the All index of the L-pool and S-pool were calculated and plotted to genome position (**Figures 3A, B**), and then Δ All index was calculated by subtracting the SNPs/indels index values of the two progenies pools (**Figure 3C**). Then, we identified the peak region above the threshold value as the candidate region for fruit length trait. With the 95% significance level, we obtained 3 QTLs located on chromosome 4, 8 and 14 (**Supplementary Table S8**). Only one InDel was found in the candidate region of chromosome 4, and another was found in the candidate region of chromosome 8. Both InDels were located in intergenic regions. Thus, two QTLs on chromosomes 4 and 8 were excluded. We also analyzed the data with QTLseqr and calculated the G’ value plotted to the genome position (**Figure 3D**). With the 95% significance level, a genome region (Cmo_Chr14: 159993–1750879) was obtained (**Supplementary Table S9**). Based on BSA results using different methods, the overlapping region (Cmo_Chr14: 718001–1711000) was referred to be the target region responsible for the length of pumpkin fruit.

### Fine−mapping of candidate gene associated with pumpkin fruit length

**Figure 4:**
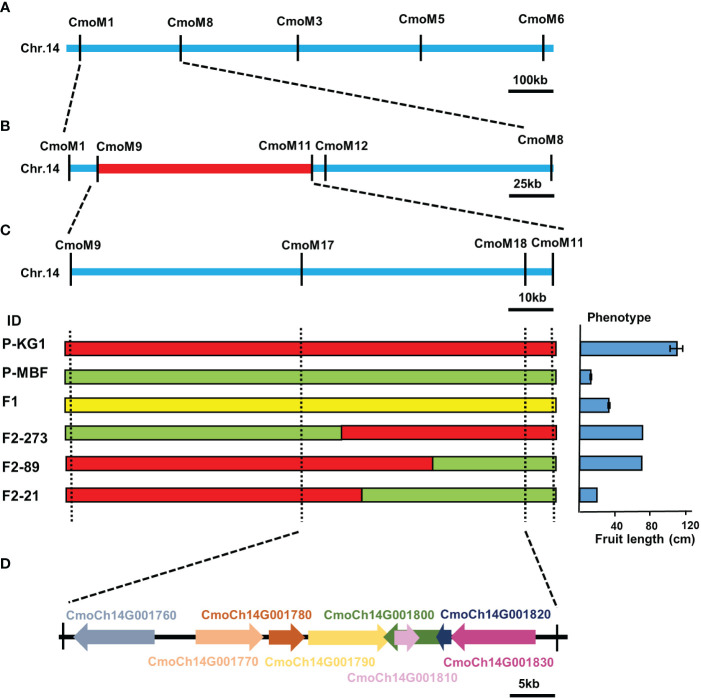
Fine mapping of the candidate gene for pumpkin fruit length. **(A)** Primary mapping of target region using 259 F_2_ individuals. The candidate gene was delimited to the region between markers CmoM1 and CmoM8. **(B)** Fine mapping of the candidate gene. The candidate gene was fine mapped in a 50.77kb region between markers CmoM17 and CmoM18. **(C)** Individuals with chromosome segment substitution on the target region, red indicates genotype of KG1, green indicates genotype of MBF, and yellow indicates heterozygous region. **(D)** In the mapping interval, there are eight genes, which may be candidate genes for target traits.

In order to confirm and narrow down the genetic interval obtained by BSA-seq analysis, KASP markers were used for fine mapping based on the initial candidate region. A total of 18 KASP markers were developed in the candidate region, and 259 F_2_ individual plants were used for screen the recombinants and phenotype-genotype association analysis. We found that F_2–_89 and F_2–_21 were genotyped identically by the CmoM17 and CmoM18 markers, but their fruit length differed. Similarly, F_2–_89 and F_2–_273 did not differ in their fruit length, but were genotyped differently using CmoM17 and CmoM18 markers. Based on the phenotype-genotype association analysis of recombinants, we finally narrowed down the target region to a 50.77 kb window between markers CmoM17 (791,681bp) - CmoM18 (842,449bp) through these three critical recombinants (**Figures 4A-C**). There were eight annotation genes in this region, *CmoCh14G001760* (Reverse transcriptase (RNA-dependent DNA polymerase), putative), *CmoCh14G001770* (Putative serine/threonine-protein kinase receptor), *CmoCh14G001780* (Nuclear transcription factor Y subunit B-8-like), *CmoCh14G001790* (E3 ubiquitin-protein ligase), *CmoCh14G001800* (DNA helicase), *CmoCh14G001810* (Pentatricopeptide repeat-containing protein, putative), *CmoCh14G001820* (DNA helicase), *CmoCh14G001830* (Small subunit rib) (**Figure 4D**; **Supplementary Table S10**). Sequence variation analysis was performed to identify the variations of these eight genes between two parents. There was no sequence variation in the coding sequences between two parents. We also analyzed the variants in the 2 kb upstream promoter region of these genes, and only *CmoCh14G001780* and *CmoCh14G001790* were found to have several variations in the promoter region co-segregating with the phenotype, indicating that these variations might regulate fruit length by affecting gene expression.

### Expression and promoter analysis of candidate gene

To further make sure which gene is responsible for the fruit length, we analyzed the expression of *CmoCh14G001780* and *CmoCh14G001790.* Since the shape of the pumpkin ovary has been formed before flowering, and a significant difference in fruit length was found between two parents at 0 DAA, we examined the expression of these two candidate genes in the ovaries at four time points (-12, -8, -4 and 0 DAA) before anthesis.

**Figure 5:**
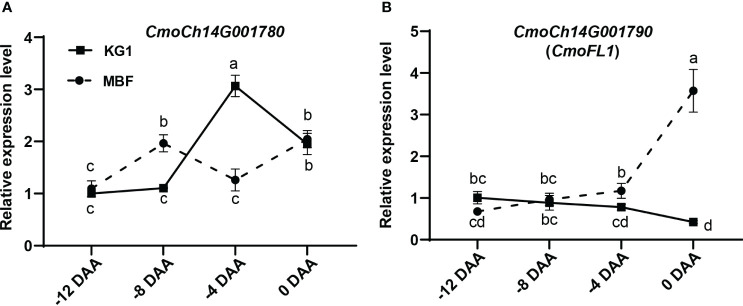
Expression analysis of candidate genes in different stages of fruit development. **(A, B)** Expression of candidate genes *CmoCh14G001780* **(A)** and *CmoCh14G001790* **(B)** during ovary development. Expression analysis suggested that *CmoCh14G001790* was the most likely candidate gene controlling pumpkin fruit length. Different letters indicated statistical significance (P < 0.05) as determined by one-way ANOVA.

Expression analysis showed that the expression pattern of *CmoCh14G001780* in both parents showed a fluctuating trend that did not correspond to the increase of fruit length (**Figure 5A**). While, the expression pattern of *CmoCh14G001790* displayed an approximately opposite trend with the development of ovary (**Figures 1C**, **5B**). The results indicated that in MBF the expression of *CmoCh14G001790* exhibited a gradual increase during the ovary development, and it showed a gradual decrease in KG1 (**Figure 5B**). Therefore, *CmoCh14G001790* was speculated as the most likely candidate gene in regulating fruit length, which may exert a negative influence on this trait. We named this gene as *CmoFL1*(*fruit length 1*), which was predicted to encode an E3 ubiquitin-protein ligase.

**Figure 6:**
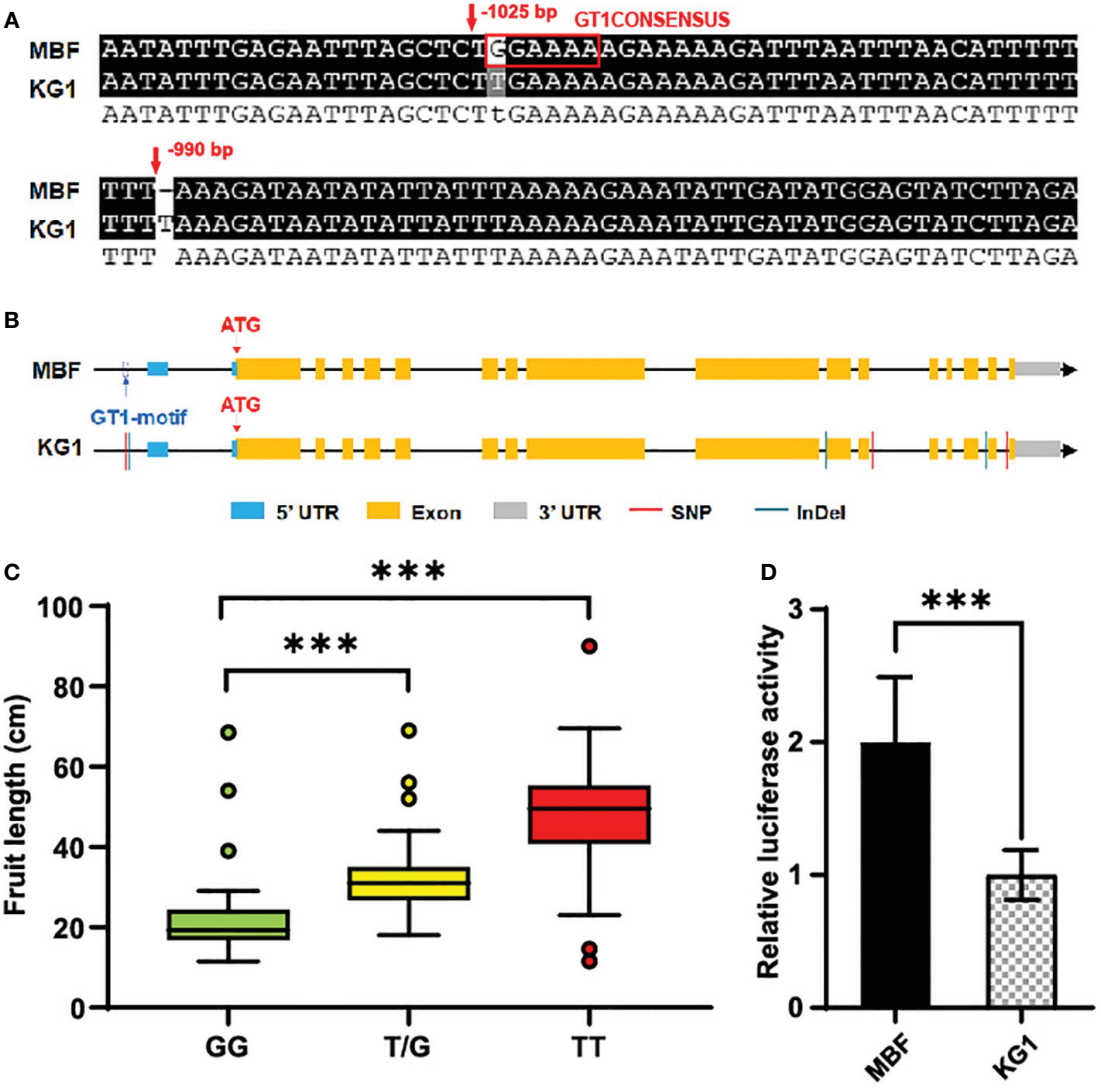
Promoter variations of candidate gene. **(A)** Partial results of sequence alignment of promoter region of *CmoFL1* between MBF and KG1. **(B)** Structure and sequence alignment of *CmoFL1* between MBF and KG1. **(C)** Association analysis between *CmoFL1* genotypes based on CmoM19 and fruit length in F_2_ individuals. GG indicates homozygous type of MBF, TT indicates homozygous type of KG1 and G/T indicates heterozygous genotypes. **(D)** DUAL-LUC assay of promoter activity in MBF and KG1. The relative value of LUC/REN indicates promoter activity. ***(P < 0.001) indicates significant difference by Student’s T-test.

Through promoter sequence analysis, we found there was a SNP and an InDel in the promoter of *CmoFL1* co-segregating with the phenotype (**Figure 6A**). The SNP in the promoter of *CmoFL1* was located in a GT1CONSENSUS (GGAAAA), which disappeared when it was mutated from G to T (**Figure 6B**). GT1CONSENSUS were reported to involved in the regulation of transcription of many genes and can act as a transcription activator or repressor (Villain et al., 1996; Buchel et al., 1999; Zhou, 1999). To further study the SNP in the promoter of *CmoFL1* and its relevance to fruit length, a KASP marker based on this SNP was developed for genotyping, named CmoM19. Among F_2_ individuals, we found that the fruit length in genotype GG (homozygous genotype with MBF) was significantly lower than TT (homozygous genotype with KG1), and the fruit length of heterozygous genotype G/T also showed significant different for that of GG (**Figure 6C**). To explore how the variations in the promoter region affect the expression of *CmoFL1*, we further analyzed the promoter activity of *CmoFL1* between two parents. We performed DUAL-LUC assay to explore whether the variation of GT1CONSENSUS affect the promoter activity. The results showed that the promoter activity of *CmoFL1* in the short fruit parent MBF was significantly higher than that in the long fruit parent KG1 (**Figure 6D**), indicating the absence of GT1CONSENSUS in KG1 could reduce the promoter activity and thus affect fruit length. Collectively, these results strongly suggested that *CmoFL1* was the candidate gene for the fruit length of pumpkin.

### Transcriptomic profiles associated with cell division

**Figure 7:**
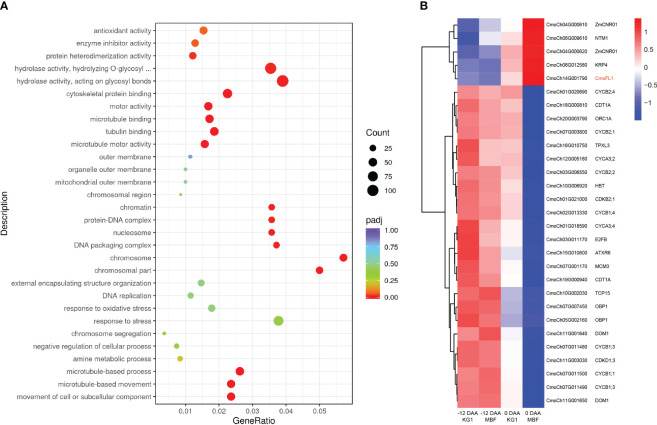
RNA-seq analysis during fruit development in long-fruit KG1 and short-fruit MBF. **(A)** GO annotation and enrichment of DEGs between KG1 and MBF at 0 DAA. **(B)** Gene expression patterns of orthologous genes involved in cell division and cell cycle in KG1 and MBF at -12 DAA and 0 DAA. Each sample has three independent biological replicates.

To explore the regulatory pathway of *CmoFL1* gene regulating the fruit length in pumpkin, we performed a transcriptome analysis during fruit development. The samples of long-fruit KG1 and short-fruit MBF at -12 DAA and 0 DAA were collected for RNA-seq analysis. In total, we obtained 76.49 Gb clean data with Q20 ≥ 97.90%, Q30 ≥ 94.07%, and a 44.78%-45.34% G/C ratio (**Supplementary Table S11**). Then, we performed differential expression analysis between KG1 and MBF pumpkins at different developmental stages. The results showed that there were 1755 (505 up-regulated and 1250 down-regulated) and 6730 (3694 up-regulated and 3036 down-regulated) differentially expressed genes (DEG) between two parents at -12 DAA and 0 DAA, respectively (**Supplementary Table S12**), indicating that the number of DEGs increased with the development of fruit length and was consistent with the phenotypic differences of fruit length development (**Figures 1B, C**). Then, Gene Ontology (GO) enrichment of the DEGs was performed between KG1 and MBF at 0 DAA. We found that the GO terms of the biological process categories “microtubule-based movement” and “microtubule-based process” and the molecular function categories “microtubule motor activity”, “tubulin binding” and “microtubule binding” were most significantly enriched at 0 DAA (**Figure 7A**). Microtubules as an important cytoskeleton were reported participate in cell growth and cell division, and were regulated by microtubule associated proteins (Kaloriti et al., 2007; Sedbrook and Kaloriti, 2008; Zeng et al., 2009; Schaefer et al., 2017). These results suggested that the transcript levels of genes associated with microtubule were different between long-fruit KG1 and short-fruit MBF at 0 DAA, indicating that the formation of fruit length may be related to the cell division process in which microtubules were involved, as the cell number of KG1 was significantly higher than that of MBF at 0 DAA (**Figure 1F**).

Cell division was a highly coordinated process in which many plant cell cycle regulators have been reported to be involved (Vandepoele et al., 2002). To determine whether *CmoFL1* involved in regulating the cell division, we analyzed the orthologous genes in *Cucurbita moschata* that have been reported to be involved in cell cycle and cell division in *Arabidopsis* or maize (**Supplementary Table S13**), and determined the gene expression patterns during fruit development. Among the DEGs, CDKs and cyclins showed higher expression levels in both parents at -12 DAA, and significantly lower expression levels in short-fruit MBF at 0 DAA, which was opposite to the expression pattern of *CmoFL1* (**Figure 7B**). The CDKs family has been reported to drive cell through cell cycle phase, and it must bind to cyclins to be activated (Vandepoele et al., 2002). Four transcription factors (*E2F*, *OBP1*, *TCP15*, *ORC1*) involved in cell cycle and their downstream genes (*MCM3*, *CDC6*, *CDT1A*) (Berckmans and De Veylder, 2009) also showed significantly lower expression levels in short-fruit MBF at 0 DAA (**Figure 7B**). Cell cycle inhibitor Kip-related protein 4 (*KRP4*) and transcription factor *NTM1* were reported as the negative regulators of cell division (Kim et al., 2006; Vieira et al., 2012), and Cell Number Regulator1 (*ZmCNR01*) in maize was identified negatively regulated cell number and organ size (Guo et al., 2010). The cluster analysis showed that these negative regulators exhibited similar expression patterns to *CmoFL1* during fruit development. They all showed lower expression levels at -12 DAA, higher expression levels at 0 DAA, and significantly higher expression in short-fruit MBF than that of long-fruit KG1 at 0 DAA (**Figure 7B**). The results indicated that *CmoFL1* may negatively regulate cell division to influence the fruit length in pumpkin.

## Discussion

Fruit length is an important crop commercial trait, which plays a vital role in fruit yield, external quality, consumer preference, packaging and transportation. The length of fruit directly affects the shape of fruit, which determines whether the fruit is oblate, round, oval, strip or other shapes, and also affects the weight of fruit, emphasizing the importance of fruit length trait in crop breeding. Previous studies have shown that in Cucurbitaceae crops, the shape of ovary at anthesis is highly correlated with the shape of mature fruit. The shape of fruit can be predicted before ovary pollination, which has been demonstrated in watermelon and melon (Dou et al., 2018; Ma et al., 2022), and a similar mechanism in pumpkin was found in this study. There was a significant correlation between FL and FSI in the parental line and F_2_ population at 0 DAA and 35 DAA (**Supplementary Table S4**), and a significant difference in ovarian length between parental lines at 8 days before anthesis (**Figures 1B, C**).

Fruit carpopodium length is an important quality trait, which affects fruit length and commercial value, and longer carpopodium will increase fruit length. Some important genes related to carpopodium length have been detected in cucumbers successively. *CsFnl7.1* encoding a late embryogenesis abundant protein, was identified to control the fruit neck length in cucumber through bulked segregant analysis (Xu et al., 2020). *Cucumis sativus HECATE1* (*CsHEC1*) was found to be highly expressed in fruit neck and interacted with CsOVATE to regulate the fruit neck length variation via modulating auxin biosynthesis (Wang et al., 2022). However, the neck length had a limited effect on the fruit length in cucumber. In pumpkin, we found that the long-fruited pumpkin KG1 has the extra-long carpopodium, while short-fruited pumpkin MBF has no carpopodium (**Figure 2A**). The correlation analysis between fruit length and carpopodium length showed a significant positive correlation between parents and F_2_ population (**Figure 2C**), suggesting that the genes associated with fruit length may also regulate the carpopodium length.

Protein ubiquitination plays key roles in multiple plant development stages and several abiotic stress responses. Importantly, diverse E3 ligases are involved in these regulatory pathways by mediating phytohormone and light signaling or other pathways (Shu and Yang, 2017). Extensive studies have shown that E3 ligases were involved in the morphological regulation of fruits and seeds. In rice, *GW2* encoding a previously unknown RING-type protein with E3 ubiquitin ligase activity, was found to play a negative role in regulating cell division in determining rice grain width and weight (Song et al., 2007). *Chang Li Geng 1* (CLG1), which encodes an E3 ligase, regulates grain size by targeting and degrading the negative grain length regulator Gγ protein GS3 (Yang et al., 2021). In wheat, *TaGW2* is an orthologue of rice gene *OsGW2*, which is also negatively regulates grain width and weight (Hong et al., 2014; Simmonds et al., 2016; Liu et al., 2019). TaSDIR1–4A was confirmed as a negative regulator of grain size. Silencing of TaSDIR1–4A (a RING-type E3 ubiquitin ligase) led to an increase in wheat TGW (1000-grain weight), while overexpression of TaSDIR1–4A in Arabidopsis resulted in a decrease in seed size (Wang et al., 2020a). In *Brassica napus*, variation in the promoter region of the HECT E3 ligase gene *BnaUPL3.C03* made a major contribution to variation in seed weight per pod (Miller et al., 2019). In addition, E3 ligases were also found to play important roles in regulating fruit size. It is reported that the RING-type E3 ligase short fruit 1 (SF1) can ubiquitinate and degrade both itself and 1-aminocyclopropane-1-carboxylate synthase 2 (ACS2) to tightly control ethylene synthesis for dose-dependent cell division and fruit elongation in cucumber (Xin et al., 2019). In addition, UV-damaged DNA binding protein 1 (DDB1), the core component of Cullin4-RING E3 ubiquitin ligase complex (CRL4), has been shown to be profoundly involved in the cell division process (Iovine et al., 2011). In the present study, we identified that *CmoFL1* encoding E3 ubiquitin ligase was a candidate gene for the fruit length of pumpkin. The expression analysis revealed that the expression pattern of *CmoFL1* in the MBF exhibited a gradual increase during ovary development, while it showed a gradual decrease in KG1 (**Figure 5B**). In addition, the result of paraffin section indicated that the cell number of KG1 fruit was significantly higher than that of MBF fruit (**Figure 1F**). RNA-seq analysis suggested that *CmoFL1* showed consistent expression patterns with cell division negative regulators (**Figure 7B**). These results suggested that high expression level of *CmoFL1* may inhibit the increase of cell number in MBF. Therefore, we speculated that the regulation pattern of *CmoFL1* in pumpkin fruit length may be similar to other E3 ligases, which negatively regulate cell division and thus affect the variation of fruit length.

In conclusion, we reported the potential candidate gene *CmoFL1* encoding E3 ubiquitin ligase was responsible for the fruit length of pumpkin. The results of this study can provide a reference for understanding the genetic mechanism of pumpkin fruit morphogenesis and will also enable marker-assisted breeding of pumpkin fruit length improvement.

## Data availability statement

The dataset presented in the study can be found in online repository (http://www.ncbi.nlm.nih.gov), accession number PRJNA1099576.

## Author contributions

YMZ: Data curation, Formal analysis, Writing – original draft. MZ: Methodology, Data curation, Writing – original draft. QS: Data curation, Formal analysis, Writing – original draft. MYZ: Data curation, Formal analysis, Writing – original draft. CW: Data curation, Formal analysis, Writing – original draft. YTZ: Data curation, Investigation, Writing – original draft. QY: Data curation, Investigation, Writing – original draft. YB: Data curation, Investigation, Writing – original draft. ZH: Data curation, Supervision, Writing – review & editing. JY: Data curation, Supervision, Writing – review & editing. MFZ: Data curation, Supervision, Writing – review & editing. XL: Data curation, Visualization, Writing – review & editing.
